# Outcomes and Determinants of Coronary Endarterectomy With Coronary Artery Bypass Graft (CABG): A Tertiary Centre’s Five-Year Experience

**DOI:** 10.7759/cureus.100061

**Published:** 2025-12-25

**Authors:** Nada Ali, Francesca Gatta, Mahmoud Elkhatib, Neeraj Mediratta

**Affiliations:** 1 Cardiothoracic Surgery, Liverpool Heart and Chest Hospital NHS Foundation Trust, Liverpool, GBR

**Keywords:** coronary artery bypass grafting (cabg), coronary endarterectomy (ce), diffuse coronary artery disease, mid-term survival, postoperative outcomes

## Abstract

Background: Coronary endarterectomy (CE) is used as an adjunct to coronary artery bypass grafting (CABG) in patients with severe diffuse coronary artery disease. The primary goal of performing CE is to achieve complete revascularisation by recreating a patent lumen when isolated CABG is not possible due to severe plaque burden. This study evaluates outcomes following CE-CABG at a tertiary centre and uniquely examines how vessel type, operative technique may influence mortality. Additionally, it highlights the heterogeneity in prescribing anti-thrombotic medications post-operatively.

Methods: We analysed urgent and elective CE-CABG procedures from 2019 to 2024, excluding patients with concomitant valve surgery or incomplete records. The primary objective was to assess short- and mid-term survival after coronary endarterectomy combined with CABG at a tertiary centre. Secondary objectives included postoperative complications, changes in heart function, and the impact of anatomical and technical factors on outcome. Survival was estimated with Kaplan-Meier analysis.

Results: The cohort comprised 48 patients (mean age 66.5 ± 9.1 years, 81.3% male), with high rates of dyslipidaemia (79.2%) and hypertension (62.5%). Most procedures were urgent (68.8%) and performed on-pump (72.9%). In-hospital mortality was 8.3%, and overall mortality during follow-up was 20.8%. Prolonged inotropic support (47.9%), extended ventilation (41.7%), and atrial fibrillation (20.8%) were the most common complications. Mortality differed by vessel: obtuse marginal (OM) endarterectomy demonstrated the highest mortality rate (42.9%), whereas left anterior descending (LAD) endarterectomy showed comparatively lower mortality (16.7%). Open endarterectomy was associated with numerically lower mortality (15%) compared with closed techniques. Considerable heterogeneity was observed in postoperative antithrombotic prescribing across the cohort. Kaplan-Meier curves demonstrated a high early postoperative risk with stable survival in the mid-term period.

Conclusions: CE-CABG carries notable early morbidity and mortality, but mid-term survival remains acceptable. This study highlights vessel- and technique-specific risk, though the limited statistical power and absence of standardised perioperative protocols constrain generalisability. Larger studies are needed to refine patient selection, optimise surgical approach, and standardise antithrombotic strategies following CE.

## Introduction

Coronary artery disease (CAD) represents a significant global health burden, necessitating effective revascularisation strategies to improve patient outcomes [1]. Coronary endarterectomy (CE) serves as a beneficial adjunct to coronary artery bypass graft (CABG) surgery, especially for achieving complete revascularisation in cases of diffuse CAD where conventional CABG techniques alone are insufficient [2,3].

While CE-CABG has been linked to increased 30-day mortality and higher rates of postoperative complications such as myocardial infarction, low cardiac output syndrome, and renal dysfunction, large meta-analyses and database studies demonstrate comparable five-year survival rates and acceptable long-term outcomes despite the higher initial mortality risk. These studies emphasize the critical role of careful patient selection [4,5].

Furthermore, individual research suggests that adverse outcomes following CE-CABG may often reflect patient comorbidities rather than the CE procedure itself. For instance, studies by Tiruvoipati et al. [6], LaPar et al. [7], and Bagheri et al. [8] indicate that CE is not an independent predictor of mortality, even though patients undergoing the procedure might present with higher baseline risks and longer ICU stays.

The primary objective of this study was to assess early and mid-term survival after coronary endarterectomy combined with coronary artery bypass grafting at a tertiary cardiothoracic centre. Secondary objectives included describing postoperative complications and changes in heart function, and investigating whether surgical factors, such as the treated artery and the operative technique, can affect outcomes. 

Our study contributes to the ongoing debate on the viability of CE-CABG for extensive CAD by reporting outcomes from our tertiary cardiothoracic centre.

## Materials and methods

This was a retrospective observational study of patients who underwent CABG-CE at Liverpool Heart and Chest Hospital NHS Foundation Trust in Liverpool, United Kingdom between August 2019 and September 2024. This study was conducted as a retrospective audit of clinical outcomes at Liverpool Heart and Chest NHS Foundation Trust and was reviewed and registered by the local clinical governance and audit department (audit ID CA100142). All patient data were anonymised before analysis.

Study population

Patients were identified through the audit team's institutional electronic coding systems, which flag endarterectomy procedures in surgical records. All cases identified through coding were subsequently verified by the authors to confirm that a coronary endarterectomy had been performed alongside CABG. All patients who underwent isolated urgent or elective CABG combined with coronary endarterectomy during the study period were included. Patients were excluded if they underwent concomitant cardiac procedures or if clinical or outcome data were incomplete.

Data collection

Clinical, operative, and outcome data were retrieved electronically by the institutional audit team and checked for completeness. Variables gathered included patient demographics and comorbidities, urgency of surgery (urgent or elective), cardiopulmonary bypass (CPB) and cross-clamp duration, number of vessels subjected to endarterectomy, the target coronary artery, and endarterectomy technique (open or closed, where documented), as well as the choice of postoperative anti-thrombotic treatment. Outcome measures comprised early (operative) mortality, postoperative complications, mid-term survival, changes in heart function, and freedom from angina. 

Follow-up

Patients were followed up in person within eight weeks of hospital discharge. Postoperative echocardiography was performed selectively based on clinical indication; analyses involving echocardiographic data were therefore limited to patients with available studies. Follow-up data were gathered through review of electronic medical records. Survival status was established from recorded follow-up encounters or death notifications. Follow-up was censored at the date of last documented clinical contact or death, whichever occurred first.

Statistical analysis

Continuous variables were reported as mean ± standard deviation (SD), and categorical variables as counts and percentages. Survival was analysed using Kaplan-Meier methods, appropriate for survival data with censored follow-up. Given the limited sample size and low number of events, adjustment for confounding using multivariable models was not feasible; therefore, subgroup analyses exploring potential mortality risk factors (including patient characteristics, urgency of surgery, target vessel, and endarterectomy technique) were descriptive. Statistical analyses were performed using R version 4.5.1 through RStudio (R Foundation for Statistical Computing, Vienna, Austria, https://www.R-project.org/) and the IBM SPSS Statistics for Windows, version 30.0 (IBM Corp., Armonk, New York, United States).

Definitions

Closed endarterectomy is performed via an arteriotomy with steady traction applied to remove the atheromatous plaque proximally and distally. Open endarterectomy involves a longitudinal arteriotomy, plaque removal under direct vision, and patch closure of the artery [9]. The choice between open and closed endarterectomy was not guided by a formal protocol and was made intra-operatively based on vessel anatomy, plaque characteristics, and surgeon judgement.

Prolonged ventilation was defined as failure to extubate within six hours postoperatively, and prolonged inotropic support as the requirement for inotropes beyond 12 hours.

Mid-term survival was defined as survival within the available follow-up period of up to five years after surgery. Early (operative) mortality was defined as any death occurring during the index hospitalisation or within 30 days of surgery, consistent with standard cardiac surgery reporting [10].

## Results

Baseline and operative characteristics 

A total of 48 patients were included in our study, with a mean follow-up time of 31.9 ± 21.8 months. As detailed in Table 1, the cohort was predominantly male (n=39, 81.3%) with a mean age of 66.5 ± 9.1 years. Patients frequently presented with common comorbidities such as dyslipidaemia (n=38, 79.2%) and hypertension (n=30, 62.5%), alongside a notable prevalence of raised BMI and smoking history. More than half of the patients (n=29, 60.4%) had a normal preoperative left ventricular ejection fraction, and most operations (n=33, 68.8%) were performed urgently.

**Table 1:** Baseline characteristics (N=48) BMI, Body mass index; IDDM, Insulin-dependent diabetes mellitus; NIDDM, non-insulin-dependent diabetes mellitus; MI, Myocardial infarction; LVEF, Left ventricular ejection

Baseline Characteristics	Frequency	Percenthage
Sex		
Male	39	81.3
Female	9	18.8
BMI ( kg/m^2^)		
Normal weight (18.5-24.9)	9	18.8
Obese (>30)	16	33.3
Overweight (25-29.9)	22	45.8
Underweight (< 18.5)	1	2.1
Diabetes		
IDDM	9	18.8
NIDDM	10	20.8
Hypertension	30	62.5
Dyslipidemia	38	79.2
Smoking Status		
Non-smoker	22	45.8
Ex-smoker	21	43.8
Current smoker	5	10.4
Prior MI < 90 days	15	31.3
Prior non-surgical intervention	7	14.6
Preoperative LVEF		
Normal (*≥* 50%)	29	60.4
Mildly reduced (41-49%)	14	29.2
Moderately to severely reduced (= 40%)	5	10.4

Regarding the surgical approach, most procedures utilised cardiopulmonary bypass (n=35, 72.9%). For these on-pump cases, the mean aortic cross-clamp time was 91.3 ± 32.1 minutes, and the cardiopulmonary bypass time averaged 136.1 ± 53.9 minutes. The majority (n=40, 83.3%) of operations employed a combination of left internal mammary artery and saphenous vein conduits, with most patients (n=25, 52.1%) receiving three grafts. 

Operative details are summarised in Tables 2, 3. In 44 patients (91.7%), endarterectomy was performed on a single vessel, with the left anterior descending (LAD) (n=18, 37.5%) and right coronary arteries (RCA) (n=12, 25.0%) being the most frequently treated. The open surgical endarterectomy technique was used in 20 cases (41.7%) and the closed technique in 11 cases (22.9%), with the operative document unclear on the remainder. Notably, only 20.8% (n = 10) of the procedures were performed on occluded vessels, as demonstrated on preoperative angiograms.

**Table 2:** Operative details Data presented as n (%) unless indicated otherwise CABG, coronary artery bypass graft; LIMA,  left internal mammary artery; SV,  saphenous vein; RA, radial artery; Xclamp, cross clamp; CPB, cardiopulmonary bypass

Operative Details	Frequency	Percentage
Operation Status		
Elective	15	31.3
Urgent	33	68.8
Cardiopulmonary Bypass		
On-pump CABG	35	72.9
Off-pump CABG	9	18.75
Conversion	4	8.3
Conduit		
Combined: LIMA + SV	40	83.3
Total arterial: LIMA+ RA	6	12.5
LIMA + RA + SV	1	2.1
LIMA Only	1	2.1
Xclamp time (min) (mean ± SD)	91.3 ± 32.1	
CPB time (min) (mean ± SD)	136.1 ± 53.9	

**Table 3:** Coronary endarterectomy details LAD, left anterior descending; OM, obtuse marginal; DIAG, diagonal; RCA, right coronary artery

Coronary endarterectomy details	Frequency	Percentage
Number of endarterectomised vessels		
Single	44	91.7
2	3	6.3
3	1	2.1
Endarterectomised Vessel		
LAD	18	37.5
OM	7	14.6
DIAG	4	8.3
Inter/ramus	2	4.2
RCA	12	25
Combined left: LAD and OM	2	4.2
Combined left and right	3	6.3
Type of Endarterectomy		
Closed	11	22.9
Open	20	41.7
Unspecified	17	35.4
CE on non-occluded vessel		
No	10	20.8
Yes	38	79.2

Postoperative and follow-up outcomes

The mean ICU stay following CE-CABG in our cohort was 6.6 ± 11.3 days, and the mean hospital stay was 19.67 ± 15.3 days.

Among the 48 patients, four (8.3%) died during the index hospitalization, and six additional patients died during the follow-up period, yielding an overall mortality of 10 patients (20.8%). Causes of in-hospital death included ventricular fibrillation (VF) arrest (n=1, 2.1%), pneumonia (n=1, 2.1%), and in two cases (n=2, 4.2%), the cause of death was undocumented, as these patients were transferred to other facilities for further treatment and rehabilitation. All deceased patients during the index hospitalization were male, above 65 years of age, and had a history of dyslipidaemia. Only one of these patients had suffered a myocardial infarction within the preceding 90 days. LVEF was normal (LVEF ≥50%) in two patients and mildly reduced (LVEF 41-49%) in the other two. All four surgical procedures were performed on an urgent basis. Each patient underwent a single-vessel endarterectomy; the LAD artery was targeted in three cases, and the OM artery in one. The technique of endarterectomy was performed by the closed method in two cases, open in one, and unspecified for the remaining patient. Furthermore, three of these patients required prolonged inotropic support exceeding 12 hours, and three necessitated ventilatory support beyond six hours postoperatively.

**Figure 1:**
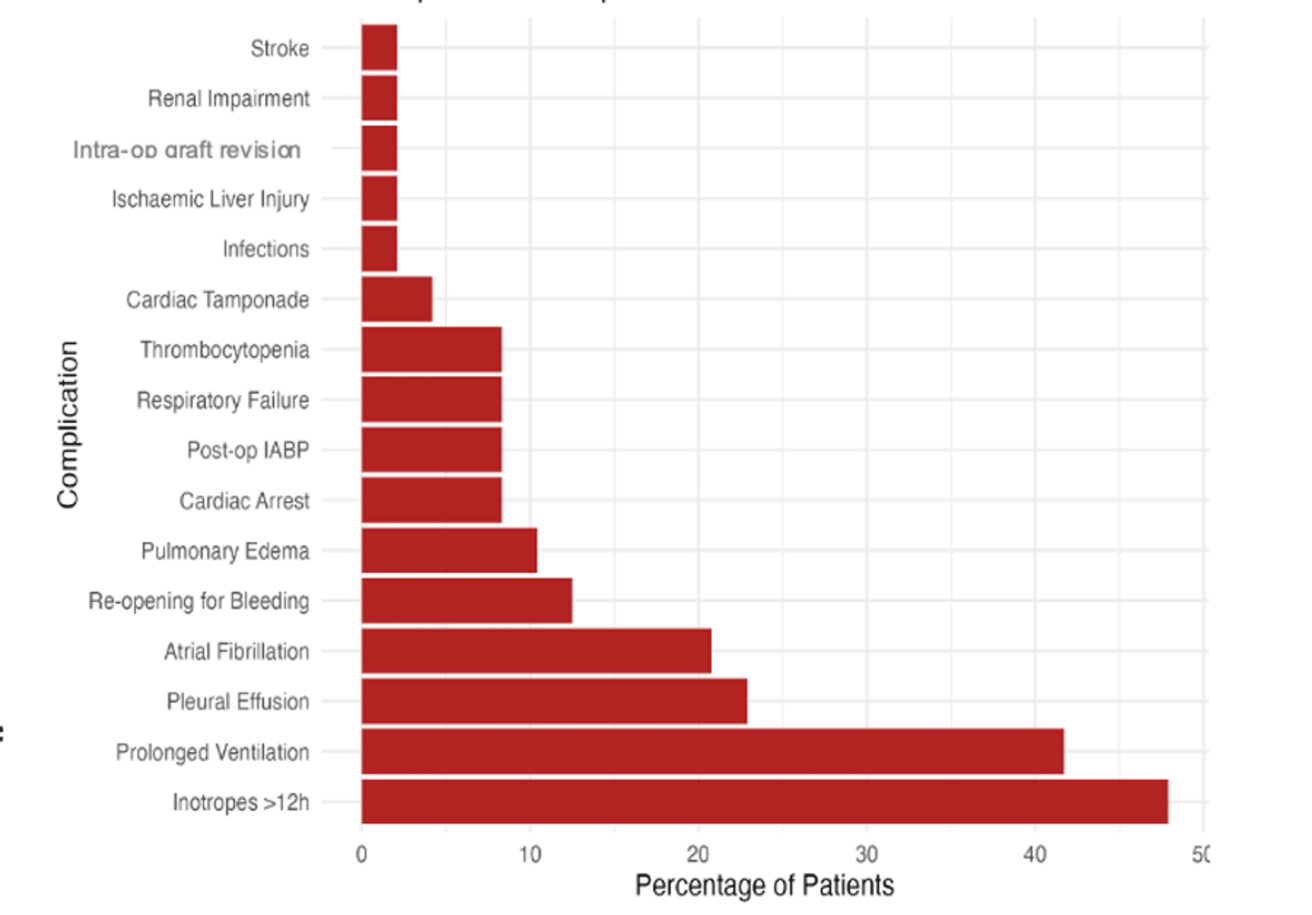
Postoperative complications IABP, intra-aortic balloon pump

Analysing the postoperative complications (Figure 1), only one patient (2.1%) required intraoperative graft revision due to ECG changes indicating ischaemia. The most common postoperative complications observed were prolonged inotropic support (n=23, 47.9%), prolonged ventilation (n=20, 41.7%), and postoperative atrial fibrillation (n=10, 20.8%).

**Figure 2:**
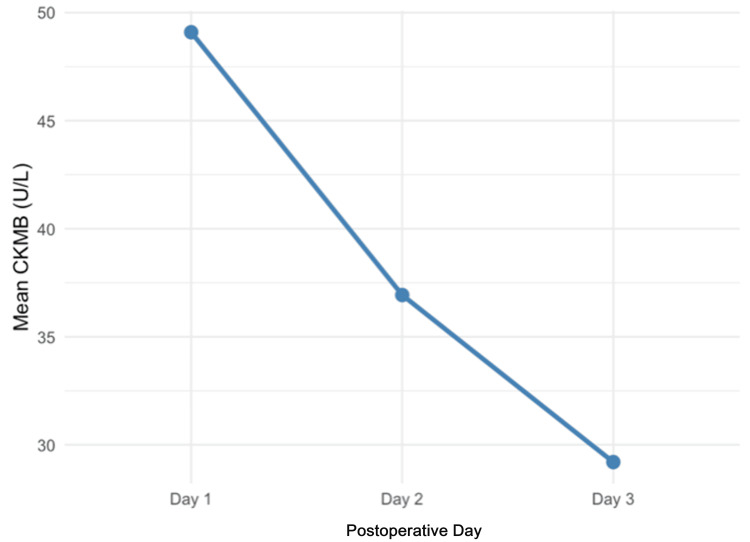
Postoperative mean CKMB levels CKMB, creatine kinase myocardial band

Postoperative cardiac enzyme levels showed the mean creatine kinase-myocardial band (CKMB) (Figure 2) level for the patient population on postoperative (POD) day 1 was 49.1 ± 63.8, which decreased to 29.2 ± 39.7 by POD 3. Postoperative echocardiograms were not available for every case. In the subgroup of patients who did have postoperative echocardiograms (n=10, 20.8%), improvement or preservation of their LVEF was noted in all but one patient.

To protect the endarterectomized vessel, practitioners had varied approaches to postoperative anticoagulation and antithrombotic therapy, reflecting a lack of standardised protocol: 43.8% (n=21) of patients received three months of direct oral anticoagulant therapy, 27.1% (n=13) received three months of warfarin, and 29.2% (n=14) received standard dual antiplatelet therapy for one year followed by lifelong aspirin.

Furthermore, a significant long-term symptomatic benefit was observed during follow-up, with 35 of the 38 patients who had available outpatient data (92.1%) remaining angina-free at a mean of 31.9 months.

Subgroup analysis

Our analysis investigated the relationship between mortality and various surgical factors associated with coronary endarterectomy. Across the entire follow-up period, 10 of 48 patients (20.8%) died, although not all deaths were directly related to the procedure. In contrast, the in-hospital mortality rate was 8.3% (n=4), as described in the previous section.

When stratified by the endarterectomized vessel, mortality varied: the highest rate (n=3, 42.9%) was observed among patients who underwent obtuse marginal artery endarterectomy, while the lowest (n=3, 16.7%) occurred in those with left anterior descending artery endarterectomy, which was also the most frequently treated vessel in our cohort.

With respect to surgical technique, the open endarterectomy method demonstrated a lower mortality rate (n=3, 15%); however, this observation warrants caution, as the operative technique was not explicitly documented in 35.4% of patients (n=17), limiting the reliability of technique-specific comparisons.

A notable finding was observed in a subgroup of patients whose preoperative angiograms showed occluded coronary arteries. In 20.8% (n=10) of the total cohort, endarterectomy was performed on such occluded vessels, and within this subgroup, no mortality was recorded during the early or mid-term postoperative phases.

**Figure 3:**
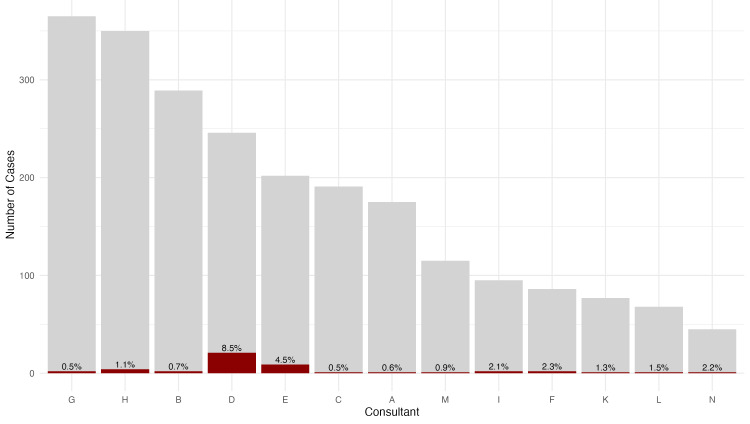
Proportion of CE-CABG procedures among total CABG cases per consultant CABG, coronary artery bypass grafting; CE, coronary endarterectomy

Lastly, we noted variability in the inclination of individual surgeons within our institution to perform coronary endarterectomies (Figure 3), with procedure rates ranging from 0% to 8.5% per operator. However, due to the limited sample size, no definitive conclusions can be drawn regarding the impact of surgeon volume or method on mortality outcomes.

Survival analysis

**Figure 4:**
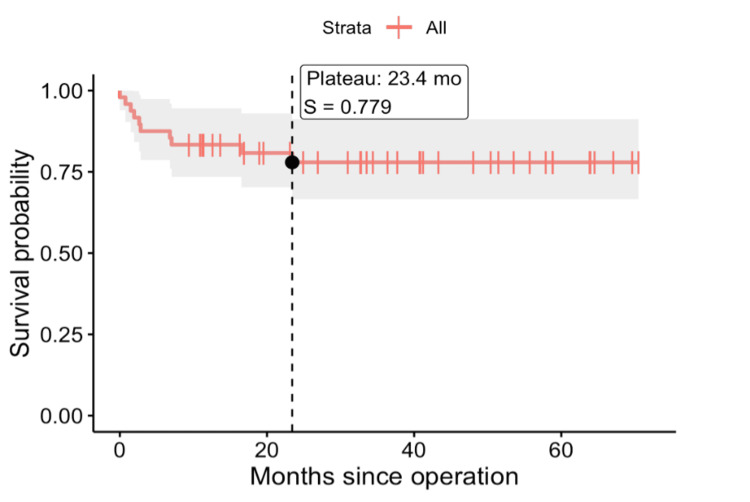
Kaplan-Meier survival curve

Kaplan-Meier survival analysis shows a high early post-operative risk, followed by stable survival in the mid-term period (Figure 4).

## Discussion

The combination of coronary endarterectomy with CABG remains a significant area of discussion in cardiac surgery, primarily aimed at achieving complete revascularisation of the myocardium in patients with extensive CAD where traditional grafting options are not feasible [9,11]. While this approach addresses a critical clinical need, reports of higher mortality and myocardial infarction rates associated with CE-CABG have sometimes led to reluctance among surgeons, potentially compromising the goal of full myocardial revascularisation [12-14].

Our study, conducted at a tertiary cardiothoracic specialist centre, aimed to evaluate the short- and mid-term outcomes of CE-CABG in a specific cohort, contributing to the ongoing discussion about its viability. The small sample size (n=48) at our centre underscores the procedure’s infrequent application, reflecting the broader diversity of clinical opinion on its effectiveness [13,15,16].

Our mortality analysis revealed an elevated early postoperative risk compared to standard CABG, aligning with findings in existing literature. The in-hospital mortality rate of 8.3% for CE-CABG in our centre is comparable with the higher early risks reported in large meta-analyses and database studies [2,4,5,14,17]. A significant proportion of these studies report operative mortality rates ranging from 1.5% to 12.0%, emphasising the variability and challenges associated with this complex procedure [18,19]. Kaplan-Meier survival analysis in our cohort similarly demonstrated a period of increased early postoperative risk, followed by relatively stable survival during mid-term follow-up. Collectively, these findings reinforce the importance of careful patient selection, surgical judgement, and perioperative management when undertaking CE-CABG.

Furthermore, the selection of the specific coronary vessel for endarterectomy and the choice between open and closed techniques are critical factors influencing patient outcomes and warrant detailed investigation. In our cohort, the left anterior descending artery was the most frequently endarterectomized vessel, reflecting its common involvement in diffuse coronary artery disease suitable for this intervention [20]. Conversely, endarterectomy of the obtuse marginal artery, while less frequent, was associated with higher mortality, suggesting a potential correlation between the anatomical location of the endarterectomized vessel and surgical risk, which warrants further investigation [21]. A possible explanation may be that the obtuse marginal vessels can be technically challenging to access, and thus difficult also to ascertain a reliably good result with the endarterectomy. The observation that open endarterectomy exhibited lower mortality rates suggests that direct visualisation and meticulous removal of atherosclerotic plaque may contribute to improved outcomes compared to blind or less direct methods. Further research is necessary to elucidate the precise mechanisms by which different endarterectomy techniques influence outcomes and to establish standardised guidelines for vessel-specific approaches [22].

Beyond mortality, our study also revealed other essential outcomes. Notably, in a selected subgroup of patients who had postoperative echocardiograms, all but one patient showed an improved or preserved LVEF compared to their preoperative status. This improvement supports the potential for CE-CABG to enhance cardiac function in patients with diffuse coronary artery disease by achieving more complete revascularisation. However, this finding is based on a limited number of patients and requires confirmation through larger studies to establish the long-term benefits of CE-CABG on ventricular function [2].

Moreover, a substantial proportion of patients with available follow-up data remained symptom-free, with 35 of 38 (92.1%) reporting no angina at a mean of 31.9 months. This finding supports the role of complete revascularisation in providing sustained symptomatic relief and underscores the importance of careful patient selection to balance these potential benefits against the inherent risks of this complex procedure [23].

A notable observation from our study was the absence of standardised guidelines for post-endarterectomy antithrombotic therapy to ensure vessel patency, as evidenced by the heterogeneous prescribing practices within our centre. While individual studies have explored differences in anticoagulation strategies post CE, current literature often suggests these differences do not significantly influence overall mortality or morbidity [2,7,14,24]. This highlights an area for future research, focusing on the efficacy and safety of various antithrombotic regimens specifically tailored for CE-CABG patients to optimise long-term graft patency and minimise thrombotic complications [25].

Limitations

This study has several limitations that should be considered when interpreting the findings. Its retrospective, single-centre design limits causal inference and introduces potential selection bias, while the relatively small sample size and low number of outcome events reduce statistical power and restrict the ability to adjust for confounding. Operative decision-making, including the choice to perform coronary endarterectomy rather than alternative strategies and the selection of open versus closed techniques, was operator-dependent and guided by intraoperative judgement, reflecting the absence of standardised protocols and limiting generalisability. In addition, postoperative echocardiography was available only for a subset of patients, consistent with routine clinical practice in a retrospective cohort; analyses involving echocardiographic data were therefore presented descriptively. The absence of a comparator group further limits conclusions regarding the relative outcomes of CE-CABG. Variability in postoperative antithrombotic therapy also precludes definitive conclusions regarding optimal management strategies. Accordingly, as an observational study, the findings should be interpreted as descriptive rather than establishing causal relationships. Despite these limitations, the study provides meaningful real-world data and highlights important considerations regarding patient selection, operative technique, and mid-term outcomes following CE-CABG.

## Conclusions

Our retrospective single-centre study demonstrates that coronary endarterectomy combined with coronary artery bypass grafting is associated with a high early postoperative risk but achieves acceptable mid-term survival in patients with extensive coronary artery disease unsuitable for conventional revascularisation. Our findings suggest that CE-CABG may improve, or at least preserve, left ventricular ejection fraction and provide effective symptomatic relief from angina; however, these observations should be interpreted cautiously given the observational design and limited sample size. Variation in postoperative antithrombotic strategies further highlights the lack of standardised guidance. Overall, these results contribute to the existing literature and support CE-CABG as a potential revascularisation option in selected patients with diffuse coronary disease, while underscoring the need for larger, comparative studies to better define optimal practice.
